# The importance of patient engagement to quality of breast cancer care and health-related quality of life: a cross-sectional study among Latina breast cancer survivors in rural and urban communities

**DOI:** 10.1186/s12905-021-01200-z

**Published:** 2021-02-09

**Authors:** Jackie Bonilla, Cristian Escalera, Jasmine Santoyo-Olsson, Cathy Samayoa, Carmen Ortiz, Anita L. Stewart, Anna María Nápoles

**Affiliations:** 1 National Institute on Minority Health and Health Disparities, National Institutes of Health, 9000 Rockville Pike, Building 3, Floor 5, Room E08, MD 20892 Bethesda, USA; 2 Division of General Internal Medicine, Department of Medicine, University of California San Francisco, 3333 California St., Suite 335, San Francisco, CA 94143-0856 USA; 3 Health Equity Research Lab, Department of Biology, San Francisco State University, 1600 Holloway Ave, San Francisco, CA 94132 USA; 4 Círculo de Vida Cancer Support and Resource Center, 2601 Mission St, Suite 702, San Francisco, CA 94110 USA; 5 Institute for Health & Aging, University of California San Francisco, 3333 California St., Suite 340, San Francisco, CA 94118 USA

**Keywords:** Patient engagement, Shared decision making, Quality of care, Quality of life, Breast cancer, Latino/hispanic

## Abstract

**Background:**

Compared to their white counterparts, Latina breast cancer survivors experience poorer quality of care and worse health-related quality of life. Limited English proficiency (LEP) and patient engagement in cancer care could help explain these disparities. We assessed associations between LEP status and difficulty engaging with physicians, with self-reported quality of breast cancer care and health-related quality of life (physical and emotional well-being) among rural and urban Latina breast cancer survivors.

**Methods:**

Analyses used cross-sectional baseline survey data from two studies that tested a stress management program among rural and urban Latina breast cancer survivors in California. Medical information was collected through medical records review. Linear regression models examined bivariate and multivariable associations of LEP status (yes or no), difficulty engaging with doctors (asking questions and participating in treatment decisions) (1–4; higher score = greater difficulty), and rural versus urban site, with three outcomes: (1) quality of breast cancer care and information; (2) physical well-being; and (3) and emotional well-being, controlling for demographic and medical factors.

**Results:**

The total sample included 304 women (151 from urban and 153 from rural sites). Mean age was 52.7 years (SD 10.9). Most were limited English proficient (84.5%) and had less than a high school education (67.8%). Difficulty engaging with doctors was inversely associated with patient ratings of quality of breast cancer care and information (B = − 0.190, *p* = 0.014), emotional well-being (B = − 1.866, *p* < 0.001), and physical well-being (B = − 1.272, *p* = 0.002), controlling for demographic and treatment factors. LEP (vs. not; B = 1.987, *p* = 0.040) was independently associated with physical well-being only. Rural/urban status was not related independently to any outcome.

**Conclusions:**

Rural and urban Latina breast cancer survivors who report greater difficulty engaging with physicians experienced worse quality of breast cancer care and health-related quality of life. Promoting greater engagement of Latina breast cancer survivors in cancer care and providing medical interpreters when needed could improve patient outcomes among this vulnerable group.

*Trial registration*: http://www.ClinicalTrials.gov identifier NCT02931552 and NCT01383174.

## Background

In 2001, the Institute of Medicine identified patient-centeredness as one of six important targets of reform for the twenty-first century U.S. health care system, simultaneously recognizing that frequently, Americans do not receive evidence-based care that meets their needs [1]. In patient-centered care, the patient’s health needs and desired outcomes drive health care decisions and quality measurement [2]. Patient engagement and collaborative shared decision making are the cornerstones of patient-centered care. There is strong evidence that both patient engagement and shared decision making are associated with better patient outcomes, including greater patient satisfaction and better physical and mental well-being [3–5]. These associations are observed for cancer patients as well. Among cancer patients, patient engagement and shared decision making are associated with greater patient satisfaction, better treatment adherence, greater self-efficacy for managing health, better quality of life, and improved survival [6–10].

Compared to white breast cancer survivors and more acculturated Latinas, less acculturated Latina breast cancer survivors report less involvement in treatment decision making, greater treatment decision making regret, less satisfaction with breast cancer care information provided, a more limited understanding of their diagnosis and treatment, lower self-efficacy for interacting with physicians, and worse patient-physician relationships [11–16]. Among Latina breast cancer survivors, greater English proficiency was associated with better communication effectiveness specific to treatment decision making, and better communication predicted greater satisfaction, which, in turn, predicted better quality of life [17]. These studies suggest that compared to their English-speaking counterparts, Spanish-speaking Latina breast cancer survivors engage less with their physicians, are less satisfied with their care, and report poorer health-related quality of life.

Patients with low English proficiency may experience or prefer more provider-driven communication because they have a harder time understanding the information provided during visits and asking for clarification [4]. In qualitative studies, Spanish-speaking Latino patients reported feeling they were a burden to physicians, preferring to get by on their limited English-speaking abilities, rather than request a medical interpreter [18].

In addition to language factors, among Latina breast cancer survivors, cultural values related to role expectations and preferred communication styles could help explain why they are less involved in their care. Traditional Latino cultural factors such as “simpatía” and respect for authority figures could help explain the lower patient activation rates of less acculturated Latinos [5, 19]. Simpatía refers to a cultural script which entails a preference for positive interpersonal relationships and avoidance of confrontation. Thus, Latinas with breast cancer may avoid asking their physicians questions, not only because of limited English fluency but also due to cultural values of reverence and respect for authority figures, such as physicians [20].

The intersectionality of being Latina and residing in a rural area has not been well-studied in the cancer survivorship literature. One study conducted among rural Latino cancer survivors found that most reported unfavorable experiences with their physicians and believed that greater access to cancer survivorship information in Spanish would be useful [21]. Lack of culturally and linguistically appropriate oncology services among rural Latino cancer survivors results in poorer communication with health care providers related to diagnosis and treatment [22]. Among rural Latino cancer survivors, these issues are further compounded by hardships related to poverty, transportation, and inadequate insurance coverage [23]. Rural Latina breast cancer patients represent an especially understudied group who may be at particularly high risk of poorer patient-physician communication and cancer outcomes.

In this study, we aimed to explore the degree to which urban and rural Latina breast cancer survivors feel engaged in their care and its potential impact on their perceived (self-reported) quality of breast cancer care and information, and breast cancer-specific quality of life. Specifically, this study examined the effects of limited English proficiency, difficulty engaging with physicians, and urban versus rural residence on quality of care and emotional and physical well-being.

## Methods

This study is a secondary data analysis of data from two randomized controlled trials (RCTs) conducted among Latina breast cancer survivors. The purpose of both studies was to test the effects of a culturally adapted 8–10 week stress management intervention on psychosocial distress and quality of life. The intervention, called *Nuevo Amanecer* (A New Dawn), was culturally tailored for urban and rural Latina breast cancer survivors, with extensive formative research and community input. The first RCT was conducted in five urban counties in Northern California: Alameda, Contra Costa, San Francisco, San Mateo, and Santa Clara [24, 25]. The second RCT was conducted in three rural counties in California: Imperial, Tulare, and Santa Cruz/Monterey (Salinas and Watsonville areas of these counties) [26]. The self-reported survey measures used to assess English fluency, patient engagement, quality of breast cancer care and information, and physical and emotional well-being were identical across studies. Detailed methods for both studies are available elsewhere [24–26]. The current analyses used aggregated baseline data from both studies.

### Sample characteristics

The sample for this analysis consists of 304 women enrolled in the two *Nuevo Amanecer* studies. Although both studies focused on Latinas with non-metastatic breast cancer and recruitment and data collection methods were identical, inclusion criteria differed slightly across studies. The first study occurred in 2011–2014 and was the first efficacy trial of the adapted intervention, so eligibility was restricted to women living in the five urban counties who had been diagnosed within the past year. For the second trial conducted in 2016–2018, the intervention was adapted based on formative work with rural Latina breast cancer survivors and community partners to be suitable also for rural Latinas regardless of time since diagnosis. In both studies, community cancer centers with close ties to oncology clinics and hospitals were the primary sources of recruitment. Community-based recruiters who worked with community organizations serving Latinos with cancer contacted women in person or on the phone, verified their eligibility, and conducted the baseline survey in person prior to randomization. The current analyses used de-identified data, therefore, was not deemed human subjects research.

### Measures

Predictors of interest included limited English proficiency (LEP), difficulty engaging with doctors, and rural/urban study site. English proficiency was assessed with the question “How well do you speak English?” with responses of: 1 = not at all, 2 = poorly, 3 = fairly well, 4 = well, 5 = very well. Participants who responded not at all, poorly, or fairly well to the English proficiency item were identified as LEP. We developed a 3-item scale assessing *difficulty engaging with doctors*. Women rated the difficulty they experienced asking doctors questions about cancer and cancer treatment, telling doctors what they want, and asking for an interpreter using a 4-level response set: 1 = not at all difficult, 2 = slightly difficult, 3 = somewhat difficult, and 4 = very difficult. We developed these items based on our prior research developing measures of the quality of interpersonal processes of care suitable for use among diverse patients, including Latinos [27, 28]. The scale was scored as the mean of non-missing items, with a possible range of 1–4, and higher scores indicating greater difficulty engaging with doctors. The Cronbach’s alpha for the *difficulty engaging with doctors* scale was 0.72 in the sample. Women from the first RCT resided in major metropolitan areas and were classified as urban, while women in the second RCT were from areas whose economies relied primarily on agribusiness and were classified as rural.

Outcomes of interest were patient-reported quality of breast cancer care/information and physical and emotional well-being. We developed a 2-item measure to assess perceived *quality of breast cancer care and information*. Women were asked to rate separately the medical care and information they received for their breast cancer using a 5-point scale: 1 = poor, 2 = fair, 3 = good, 4 = very good, and 5 = excellent. The 2-item scale was scored as the mean of non-missing items, with a possible range of 1–5, and higher scores indicating higher quality of care. The Cronbach’s alpha for the *quality of breast cancer care and information* scale was 0.94.

We used the Functional Assessment of Cancer Therapy-Breast (FACT-B) scales as breast cancer-specific quality of life measures assessing physical and emotional well-being, which were available in Spanish [29]. These scales are common in cancer research and well-validated and were scored per the developer’s instructions. The Emotional Well-Being Scale Score ranged from 0 to 20. Cronbach’s alpha for the Emotional Well-Being Scale was 0.87. The Physical Well-Being Scale Score ranged from 0 to 24. Cronbach’s alpha for the Physical Well-Being scale was 0.90. For both scales, a higher score indicates better well-being.

In both studies, breast cancer diagnostic and treatment information were collected using similar chart review abstraction methods. These measures, which served as covariates, included age at baseline (continuous), years since initial diagnosis (< 1 year, 1–5 years, or > 5 years), education level (less than high school, completed high school, more than high school), breast cancer stage at diagnosis (stage 0, stage I, stage II, or stage III), surgery type (breast-conserving surgery, mastectomy, or none), and treatment type (both chemotherapy and radiation, only chemotherapy, only radiation, or none).

### Statistical analysis

Descriptive statistics were used to characterize the sample and test for rural–urban differences in sample characteristics. Bivariate and multivariable linear regression models were used to assess the effects of limited English proficiency (yes versus no), *difficulty engaging with doctors* (continuous), and urbanicity (rural versus urban) on each of the three outcomes of *quality of breast cancer care and information* (continuous), emotional well-being (continuous), and physical well-being (continuous), controlling for demographic and breast cancer-related characteristics.

## Results

### Sample

The total sample consisted of 304 Latina breast cancer survivors. Mean age of participants was 52.7 (SD, 10.9) years (Table 1). The majority of participants were within 1 year of diagnosis (66.1%). Most participants had less than a high school education (67.8%). About 85% of women had limited English proficiency. About 60% of women were diagnosed with stage I or II invasive breast cancer. More women had undergone breast-conserving surgery (52.6%) than mastectomy (45.7%). About half of women had received both radiation and chemotherapy (49.7%).

**Table 1:** Sample characteristics of rural and urban Latina breast cancer survivors, Nuevo Amanecer I and II, N = 304

	Total sample n = 304	Urban n = 151	Rural n = 153	*p*
Age in years, mean (SD)	52.7 (10.9)	50.5 (10.9)	54.8 (10.4)	**<** **0.001**^e^
Years since diagnosis; n (%)				**< 0.001**^e^
< 1 year	201 (66.1)	151 (100)	50 (32.7)	
1–5 years	78 (25.7)	0 (0)	78 (51)	
> 5 years	25 (8.2)	0 (0)	25 (16.3)	
Education, n (%)				0.233
Less than high school	206 (67.8)	100 (66.2)	106 (69.3)	
High school	44 (14.5)	27 (17.9)	17 (11.1)	
More than high school	53 (17.4)	24 (15.9)	29 (19.0)	
Missing	1 (0.3)	0 (0)	1 (0.7)	
Limited English proficiency, n (%)				0.115
Yes	257 (84.5)	133 (88.1)	124 (81.0)	
No	46 (15.1)	18 (11.9)	28 (18.3)	
Missing	1 (0.3)	0 (0)	1 (0.7)	
Breast cancer stage at diagnosis, n (%)				**<** **0.001**^e^
0	48 (15.8)	40 (26.5)	8 (5.2)	
I	68 (22.4)	23 (15.2)	45 (29.4)	
II	112 (36.8)	57 (37.7)	55 (35.9)	
III	58 (19.1)	31 (20.5)	27 (17.6)	
Missing	18 (5.9)	0 (0)	18 (11.8)	
Surgery type, n (%)				0.457
Breast conserving surgery	160 (52.6)	84 (55.6)	76 (49.7)	
Mastectomy	139 (45.7)	67 (44.4)	72 (47.1)	
None	1 (0.3)	0 (0)	1 (0.7)	
Missing	4 (1.3)	0 (0)	4 (2.6)	
Adjuvant treatment, n (%)				**0.002**^e^
Both radiation and chemotherapy	151 (49.7)	60 (39.7)	91 (59.5)	
Only chemotherapy	47 (15.5)	25 (16.6)	22 (14.4)	
Only radiation	70 (23.0)	42 (27.8)	28 (18.3)	
No treatment	34 (11.2)	24 (15.9)	10 (6.5)	
Missing	2 (0.7)	0 (0)	2 (1.3)	
Difficulty engaging with doctors^a^, mean (SD)	1.7 (0.8)	1.8 (0.8)	1.6 (0.8)	**0.032**^e^
Quality of breast cancer care and information^b^, mean (SD)	4.0 (0.9)	4.1 (1.0)	4.0 (0.9)	0.670
Emotional well-being^c^; mean (SD)	13.4 (4.8)	12.5 (5.0)	14.4 (4.3)	< **0.001**^e^
Physical well-being^d^; mean (SD)	16.7 (5.4)	16.0 (5.5)	17.4 (5.3)	**0.030**^e^

^a^Difficulty engaging with doctors scale, 3-item scale with response options of 1 = not at all difficult to 4 = very difficult; higher score = greater difficulty; scale score = mean of non-missing values; range = 1–4

^b^Quality of breast cancer care and information, 2-item scale with response options of 1 = poor to 5 = excellent; higher score = more satisfied; scale score = mean of non-missing values; range = 1–5

^c^Emotional well-being, 5-item scale with response options of 0 = not at all to 4 = very much; higher score = greater difficulty; scale score = mean of non-missing values; range = 0–20

^d^Physical well-being Scale, 6-item scale with response options of 0 = not at all to 4 = very much; higher scores = greater difficulty; scale score = mean of non-missing values; range = 0–24

^e^Bolded font indicates p-values that were statistically significant at p < 0.05 or less

Rural women tended to be older than urban women (mean age of 54.8 years versus 50.5 years; *p* < 0.001). Years since initial diagnosis also differed by design (eligibility in the first study was restricted to less than one year since diagnosis), with 32.7% of rural women versus 100% of urban women being recruited within less than a year since diagnosis (p < 0.001). Rural women were less likely than urban women to be diagnosed at stage 0 (in situ*)* (5.2% versus 26.5%, *p* < 0.001) and to receive both radiation and chemotherapy (59.5% versus 39.7%, p < 0.01). Rural women reported less difficulty engaging with doctors than urban women (mean = 1.6 versus 1.8, *p* < 0.05) and better emotional (mean = 14.4 versus 12.5, *p* < 0.001) and physical well-being (mean = 17.4 versus 16.0, *p* < 0.05).

### Bivariate linear regression analyses

In the bivariate models, greater difficulty engaging with doctors was significantly associated with lower quality of breast cancer care (B = − 0.165, *p* < 0.05), and worse emotional (B = − 1.913, *p* < 0.001) and physical well-being (B = − 1.312, *p* < 0.01). Compared to urban women, women from rural sites reported better emotional (B = 1.925, *p* < 0.001) and physical (B = 1.349, *p* < 0.05) well-being (Table 2).

**Table 2:** Correlates of breast cancer care quality and physical and emotional well-being, Nuevo Amanecer I and II, N = 304

	Quality of breast cancer care				Emotional well-being				Physical well-being			
	Bivariate model		Multivariate model		Bivariate model		Multivariate model		Bivariate model		Multivariate model	
	B	*p* value	B	*p* value	B	*p* value	B	*p* value	B	*p*-value	B	*p* value
	R^2^ = 0.0548				R^2^ = 0.2075				R^2^ = 0.1638			
Limited English proficiency												
No	REF		REF		REF		REF		REF		REF	
Yes	0.150	0.323	0.178	0.328	− 0.531	0.488	0.055	0.948	1.330	0.126	1.987	**0.040**^a^
Difficulty engaging with doctors	− 0.165	**0.015**^a^	− 0.190	**0.014**^a^	− 1.913	**<** **0.001**^a^	− 1.866	**<** **0.001**^a^	− 1.312	**0.001**^a^	− 1.272	**0.002**^a^
Urbanicity												
Urban	REF		REF		REF		REF		REF		REF	
Rural	− 0.046	0.670	− 0.105	0.544	1.925	**<** **0.001**^a^	1.547	0.052	1.349	**0.030**^a^	0.789	0.389
Age at baseline	− 0.002	0.629	− 0.007	0.221	0.057	**0.022**^a^	0.014	0.620	0.049	0.086	− 0.010	0.744
Years since initial diagnosis												
< 1 year	REF		REF		REF		REF		REF		REF	
1–5 years	− 0.027	0.832	− 0.009	0.962	1.954	**0.002**^a^	0.418	0.634	2.109	**0.003**^a^	1.596	0.116
> 5 years	0.000	0.999	0.236	0.392	2.301	**0.021**^a^	1.491	0.237	2.009	0.077	1.991	0.171
Education												
More than high school	REF		REF		REF		REF		REF		REF	
Less than high school	0.041	0.780	0.023	0.890	− 0.136	0.853	0.137	0.859	0.099	0.906	0.035	0.969
High school	0.078	0.690	0.068	0.746	1.759	0.071	2.302	**0.018**^a^	− 0.132	0.905	0.273	0.806
Stage												
0	REF		REF		REF		REF		REF		REF	
I	− 0.191	0.295	− 0.260	0.246	− 0.403	0.657	− 0.729	0.476	− 0.090	0.928	− 1.187	0.315
II	− 0.211	0.204	− 0.295	0.190	− 0.454	0.585	− 0.364	0.723	− 1.925	**0.036**^a^	− 2.352	**0.048**^a^
III	− 0.276	0.144	− 0.324	0.199	− 0.926	0.325	0.005	0.997	− 2.874	**0.006**^a^	− 2.060	0.122
Surgery												
Breast conserving surgery	REF		REF		REF		REF		REF		REF	
Mastectomy	− 0.042	0.703	0.020	0.883	0.040	0.942	− 0.267	0.671	− 0.854	0.174	− 0.755	0.298
None	0.447	0.642	0.594	0.558	− 11.43	**0.017**^a^	− 12.205	**0.009**^a^	− 11.11	**0.041**^a^	− 12.766	**0.018**^a^
Adjuvant breast cancer treatment												
No treatment	REF		REF		REF		REF		REF		REF	
Both chemotherapy and radiation	0.107	0.552	0.251	0.291	− 0.976	0.284	− 1.466	0.179	− 1.629	0.111	− 1.366	0.278
Only chemotherapy	0.253	0.236	0.341	0.181	− 1.099	0.309	− 1.025	0.380	0.387	0.749	1.474	0.274
Only radiation	0.376	0.059	0.337	0.134	0.026	0.979	− 0.327	0.750	0.167	0.882	− 0.493	0.678

^a^Bolded font indicates p-values that were statistically significant at p < 0.05 or less

Of the covariates, compared to younger women, older women reported better emotional well-being only (B = 0.057, *p* < 0.05). Both years since diagnosis and surgery type were significantly associated with better emotional well-being. Compared to those diagnosed within less than a year, those diagnosed within 1–5 years (B = 1.954, *p* < 0.01) and more than five years (B = 2.301, *p* < 0.05) reported better emotional well-being. Compared to those diagnosed within less than a year, women diagnosed within 1–5 years reported better physical well-being (B = 2.109, *p* < 0.01). Compared to those diagnosed at stage 0, women diagnosed at stage II (B = − 1.925, *p* < 0.05) or stage III (B = − 2.874, *p* < 0.01) were more likely to report worse physical well-being.

### Multivariable linear regression analyses

In the multivariable models, compared to those who did not have LEP, women who had LEP reported better physical well-being (B = 1.987, *p* < 0.05). Difficulty engaging with doctors was significantly associated with all three outcomes. Greater difficulty engaging with doctors was associated with lower quality of breast cancer care (B = − 0.190, *p* < 0.05), and worse emotional (B = − 1.866, *p* < 0.001) and physical well-being (B = − 1.272, *p* < 0.01). Urban/rural residence was not associated independently with any of the outcomes (Table 2).

Of the covariates, compared to those with more than a high school level education, those with high school level education reported better emotional well-being only (B = 2.302, *p* < 0.05). Women diagnosed at stage II were less likely than those diagnosed at stage 0 to report worse physical well-being (B = − 2.352, *p* < 0.05). Compared to women receiving breast-conserving surgery, those receiving no surgery were more likely to report worse emotional (B = − 12.205, p < 0.01) and physical well-being (B = − 12.766, *p* < 0.05) (Table 2).

## Discussion

This study sought to determine if limited English proficiency and difficulty engaging with physicians were associated with quality of care for breast cancer and emotional and physical well-being among rural and urban Latina breast cancer survivors. We found that Latina breast cancer survivors who reported greater difficulty engaging with physicians were more likely to report lower quality of care and information for breast cancer and poorer emotional and physical well-being, controlling for other factors. There were no differences between rural and urban Latinas in any of these outcomes. Thus, the extent of patient involvement in care was a robust correlate of patient outcomes among this vulnerable group, regardless of rural or urban residence. Limited English proficiency was associated with physical well-being only, independent of difficulty engaging with physicians and demographic and breast cancer characteristics.

Our finding that difficulty engaging with physicians was inversely associated with emotional and physical well-being indicates that health-related quality of life is negatively affected by the inability of Latina breast cancer survivors to engage with their cancer care providers. This is especially problematic among Spanish-speaking Latinas; they report the greatest desire for involvement in decision making or more information yet report less participatory decision making and information compared to their English-speaking Latina or white counterparts [30, 31]. Thus, poorer physician–patient communication most likely drives the lower quality of breast cancer care ratings found among Latinas facing a breast cancer diagnosis because it results in a poorer understanding of the diagnosis and treatment plan [31–33]. Prior studies have demonstrated that Latinas experience worse quality of life after breast cancer than their white counterparts [13, 34], and lack of culturally and linguistically appropriate cancer care and information are likely contributors [17, 30].

The finding that LEP status was independently associated with physical well-being only is probably due to its operating as a surrogate measure for other social determinants of health, independent of experiences of difficulties with patient-physician communication. The measure of LEP needs to be deconstructed with respect to associations of its components with processes and outcomes of health care. Also, we may not have found a relationship between LEP status and ratings of the quality of breast cancer care due to limited variation on English-speaking ability in our sample.

Some of the findings with respect to the bivariate comparisons of urban and rural Latinas merit further study. Rural women were less likely than urban women to be diagnosed at stage 0 (in situ*)* and more likely to receive both radiation and chemotherapy. Rural Latinas may have more limited access to care, which contributes to being diagnosed with more advanced disease. In one of the few population-based studies examining adequacy of cancer care by race/ethnicity and rural/urban residence, rural Latino cancer patients reported worse access to needed cancer care than their urban counterparts [35]. Also, of note in our study, rural women reported less difficulty engaging with physicians than urban women and better emotional and physical well-being. It could be that in rural areas, smaller communities mean better relationships between women and their health care providers who may be more sensitive to their unique needs or more fluent in Spanish. Better health-related quality of life among rural women might indicate greater resiliency or stronger social networks in rural environments where residents may be more interdependent than in urban settings. These findings present interesting questions for future research.

Compared to white breast cancer patients, those who are of racial and ethnic minority backgrounds are at a disadvantage when it comes to shared decision making during clinical encounters due to poorer communication and less relationship-building efforts of physicians [36]. Furthermore, minority patients, including Latinos, tend to be seen in safety-net settings where physicians tend to report greater contextual barriers of limited time and resources and overwhelming content of visits [37]. Yet these groups continue to be underrepresented in studies that can lead to improvements in health care processes and outcomes. A systematic review examining shared decision making among minority patients in the U.S. concluded that despite strong policy initiatives and evidence of the advantages of shared decision making, there is a lack of representation of minority populations in these studies [4]. Furthermore, most studies that have addressed cancer in rural communities have focused on utilization of cancer screening only.

Several study limitations are to be noted. The first trial of *Nuevo Amanecer* only included women who were within the first year of diagnosis, but the second trial of *Nuevo Amanecer* also included longer-term survivors, thus, unmeasured sources of bias may have been introduced by the variation in this eligibility criterion. However, including both short- and long-term survivors in the present analyses may have increased the generalizability of our findings. Both samples were largely composed of Latinas who were of Mexican origin and Spanish-speaking primarily, therefore, results may not generalize to other national origin groups or Latinas with greater fluency in English.

## Conclusions

Our study calls attention to the importance of facilitating communication between Latina breast cancer survivors and their physicians, and the link between patient engagement in care, quality of breast cancer care, and health-related quality of life among this vulnerable population. Promoting greater engagement of Latina breast cancer survivors in cancer care and providing professional medical interpreters when needed could improve patient outcomes among this vulnerable group. Clinicians need to proactively elicit patients’ concerns and preferences among Latina breast cancer patients. Health equity with respect to engagement in cancer care is a critical issue among Latina breast cancer survivors and other minorities that deserves greater scientific inquiry to guide the development of evidence-based interventions.

## Data Availability

The datasets used and analyzed in the current study are available from the corresponding author on reasonable request and appropriate data use agreements.

## Abbreviations

LEP: Limited English proficiency
RCT: Randomized controlled trial
